# Beyond Water Content: Unraveling Stiffness in Hydrated Materials by a Correlative Brillouin–Raman Approach

**DOI:** 10.1021/acsphotonics.5c00808

**Published:** 2025-06-21

**Authors:** Alessandra Anna Passeri, Francesco Morena, Chiara Argentati, Francesco Bonacci, Igor Neri, Daniele Fioretto, Massimo Vassalli, Sabata Martino, Maurizio Mattarelli, Silvia Caponi

**Affiliations:** † Dip. di Fisica e Geologia, Università di Perugia, Via A. Pascoli, Perugia 06123, Italy; ‡ Dip. di Chimica, Biologia e Biotecnologie, Università di Perugia, Via del Giochetto, Perugia 06122, Italy; § J. Watt School of Engineering, University of Glasgow, Glasgow G12 8QQ, United Kingdom; ∥ CNR - Istituto Officina dei Materiali (IOM), Via A. Pascoli, Perugia 06123, Italy

**Keywords:** Brillouin microscopy, Raman spectroscopy, mechanical properties, mechanobiology

## Abstract

Brillouin microscopy is revolutionizing bioimaging by enabling noninvasive, label-free mapping of the microscale mechanical properties of biological samples. However, the lack of a robust physical model to disentangle the significant influence of refractive index, density, and, most critically, water content on the Brillouin signal limits its applicability and widespread adoption in complex heterogeneous materials. To address this limitation, a novel Brillouin–Raman correlative microscopy approach is proposed and demonstrated on single cells. In particular, the effects of paraformaldehyde fixation on the morphological, mechanical, and chemical properties of HEK293T cells were investigated. Following fixation, an unexpected decrease in stiffness was observed, accompanied by compositional changes detected via Raman spectroscopy. By modeling cells as biphasic systems, consisting of water and dry mass, the hydration could be decoupled from the stiffness measurements. This approach represents a significant advance in biomechanical analysis, enabling the reliable interpretation of Brillouin data and facilitating the three-dimensional micromechanical characterization of biological materials. Furthermore, it has wide-ranging potential applications in biological research, particularly in contexts where hydration plays a fundamental role, paving the way for novel insights into the diagnosis and analysis of biomedical samples.

## Graphical abstract

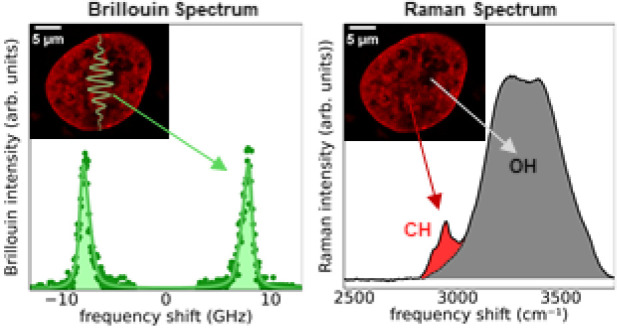

## 1 Introduction

In recent years, the interest of the scientific community in investigating the mechanobiology of living 3D samples has sparked the development of innovative elastography methods with increasing resolution and penetration depth. In this context, photoacoustic methods have undergone significant development, obtaining tomographic reconstruction, even in vivo, with resolution down to single cells.^1–3^ Among the various measurement strategies based on the interaction of light and acoustic waves, Brillouin microscopy is emerging as a novel approach that measures mechanical properties through the inelastic scattering process between photons and spontaneous high-frequency phonons present in materials.^4,5^ The technique combines the high spatial resolution of light microscopy with the noninvasive and label-free characteristics of acoustics, enabling mechanical imaging at the subcellular level.^6–8^ With increasing recognition of mechanobiology, Brillouin imaging is poised to make a significant impact on biomedical research: the technique has already demonstrated its ability to analyze , monitor, and understand the development of physiological and pathological processes at the single cell and tissue level.^9–17^

Despite its great potential, there are some open questions regarding the use of the information contained in the Brillouin spectrum. In particular, knowledge of the local refractive index and density is necessary to extract elastic moduli from the position of the Brillouin peak. Moreover, and even more importantly, the influence of water on the mechanical properties extracted from Brillouin spectra has ignited an intense debate.^18–20^ The role of water in the mechanical properties of hydrated materials is also a major source of discrepancy between measurements performed with Brillouin and other methods, such as Atomic Force Microscopy (AFM).^5^ In fact, in some cases, a positive correlation has been demonstrated between the longitudinal modulus (*M*) measured with Brillouin and the Young’s modulus (*E*) measured with indentation devices^21^ but this relationship is fundamentally sample-dependent.

To shed light on these critical issues, we developed an all-optical correlative method capable of accurately estimating the refractive index, density, and local water content in the sample. The method can be applied to a wide number of samples and provides accurate extraction of the elastic moduli by isolating changes due to water concentration. To demonstrate its potential, we applied the method to the analysis of a cellular sample before and after fixation. The study of chemical fixation not only offers a controlled process for testing the validity of the decoupling method but also addresses the general question of the impact of fixation on the mechanical properties of biological samples. In order to determine the modifications in the longitudinal elastic modulus of cells induced by the fixation protocol, the data from simultaneous Brillouin and Raman microscopy^22^ were combined with 3D fluorescence and phase holographic imaging. Their integration allowed for a comprehensive assessment of the mechanical and biochemical modifications at the single-cell level.

### 1.1 Theoretical Background: From the Brillouin Frequency Shift to the Longitudinal Elastic Modulus

Brillouin spectroscopy is based on the analysis of the frequency shift of an incoming laser beam of wavelength λ, which interacts with the internal vibrational modes of the samples. In a backscattering configuration, the Brillouin spectrum appears as a triplet, consisting of two peaks (referred to as Stokes and anti-Stokes Brillouin peaks) equally shifted by a frequency ω_*B*_ from the central Rayleigh line, which contains the elastically scattered light. Viscoelastic information can be extracted from the Brillouin spectrum.^4^ In particular, the longitudinal elastic modulus, *M*, is proportional to $\omega_{B}^{2}$ by

1 $$M=\frac{\omega_{B}^{2}\lambda^{2}}{4}\frac{\rho }{n^{2}}$$

To obtain *M*, the refractive index, *n*, and the mass density, ρ, are required, but their values are not trivially available in heterogeneous, complex samples. In some cases, the literature assumes *n* ^2^ and ρ to be proportional because both typically scale with local electron density. Using this approximation, it is justified to refer to the Brillouin frequency shift as an indicator of mechanical properties, but in some cases, as it occurs in lipid droplets of adipocytes, this approximation fails.^23^

Therefore, to obtain accurate information about the longitudinal elastic modulus as measured by Brillouin spectroscopy, it is essential to know the local refractive index and mass density. The Clausius–Mossotti relation, or the equivalent Lorentz–Lorenz formula, establishes that refractive index and density are linked to each other through the electronic polarizability of the material’s constituents (atoms and/or molecules).

2 $$\frac{n^{2}-1}{n^{2}+2}=\frac{4\pi }{3}N\alpha$$

where *N* is the number density of the molecules, and α their polarizability. This relation can also be extended to complex samples, which can be modeled as a mixture of two or more species. Assuming that the polarizability of the molecules remains unchanged upon mixing, the refractive index can be estimated as the sum of the molecular polarizability contributions from each species, weighted by their relative concentrations.^24^ Within this framework, the problem shifts to determining the biochemical composition of the sample, a challenge that can be addressed using complementary techniques such as mass spectrometry or Raman microscopy.^23,25^ Here, we present a robust methodology based on correlative Brillouin and Raman spectroscopy. In a more general framework, the method demonstrates the importance of a correlative investigation for a more profound mechanical characterization of biological materials.

## 2 Results and Discussion

### 2.1 Correlative Brillouin and Raman Microspectroscopy (BRMS)

Brillouin spectroscopy measurements in live and fixed HEK293T cells were performed, probing different subcellular structures: the nucleus, cytoplasm, and nucleoli (Figure 1a,b). The obtained Brillouin frequency shift, ω_*B*_, and Brillouin width, Γ, are reported in Figure 1c,d, revealing distinct variations among these structures. Interestingly, the most striking and unexpected finding is the global reduction in both frequency shift and width following fixation: ω_*B*_, commonly considered a proxy for stiffness, reveals a surprising global softening of cells after fixation. Leveraging the capabilities of our experimental setup, which enables the simultaneous detection of Brillouin and Raman signals, we analyzed the corresponding changes in Raman spectra. The raw data shown in Figure 2a depict the high-frequency region of the spectrum. This region features the CH_2_ and CH_3_ stretching vibrations, centered at around 2800 cm^–1^, alongside the characteristic OH stretching band, centered at around 3300 cm^–1^. While the intensity of the OH band is directly linked to water content—including both bound (hydration) water and bulk (free) water—the CH bonds are nonspecific and represent all the organic material within the cell.^26,27^ The intensity of the CH band is here proposed as a proxy for the cell dry mass.

**Figure 1:**
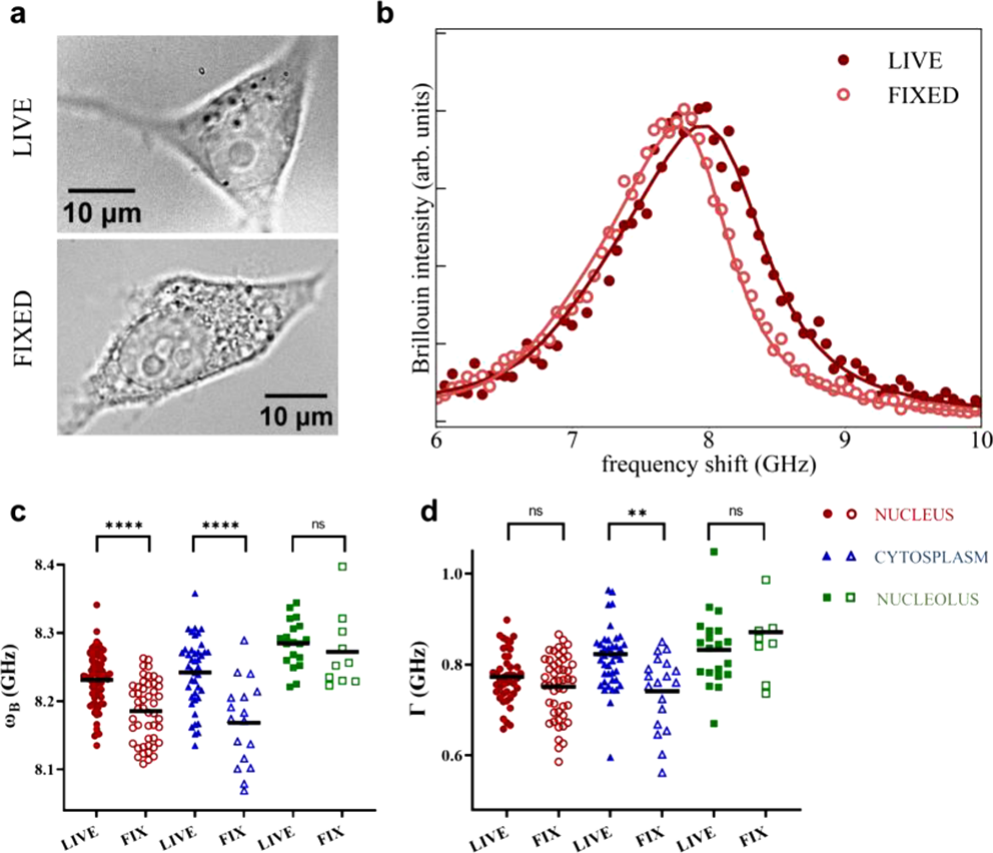
a) Representative bright-field images of live and fixed HEK cells. b) Representative Brillouin spectra for live and fixed cells. c,d) Scatter plots showing frequency shift and peak width extracted from Brillouin spectra, acquired on live and fixed cells. Data for nucleus, cytoplasm, and nucleoli are displayed in different colors. The data distributions have been compared in pairs with an unpaired two-tail *t*-test, and *p* ≤ 0.05 was considered statistically significant (GraphPad 4.0).

**Figure 2:**
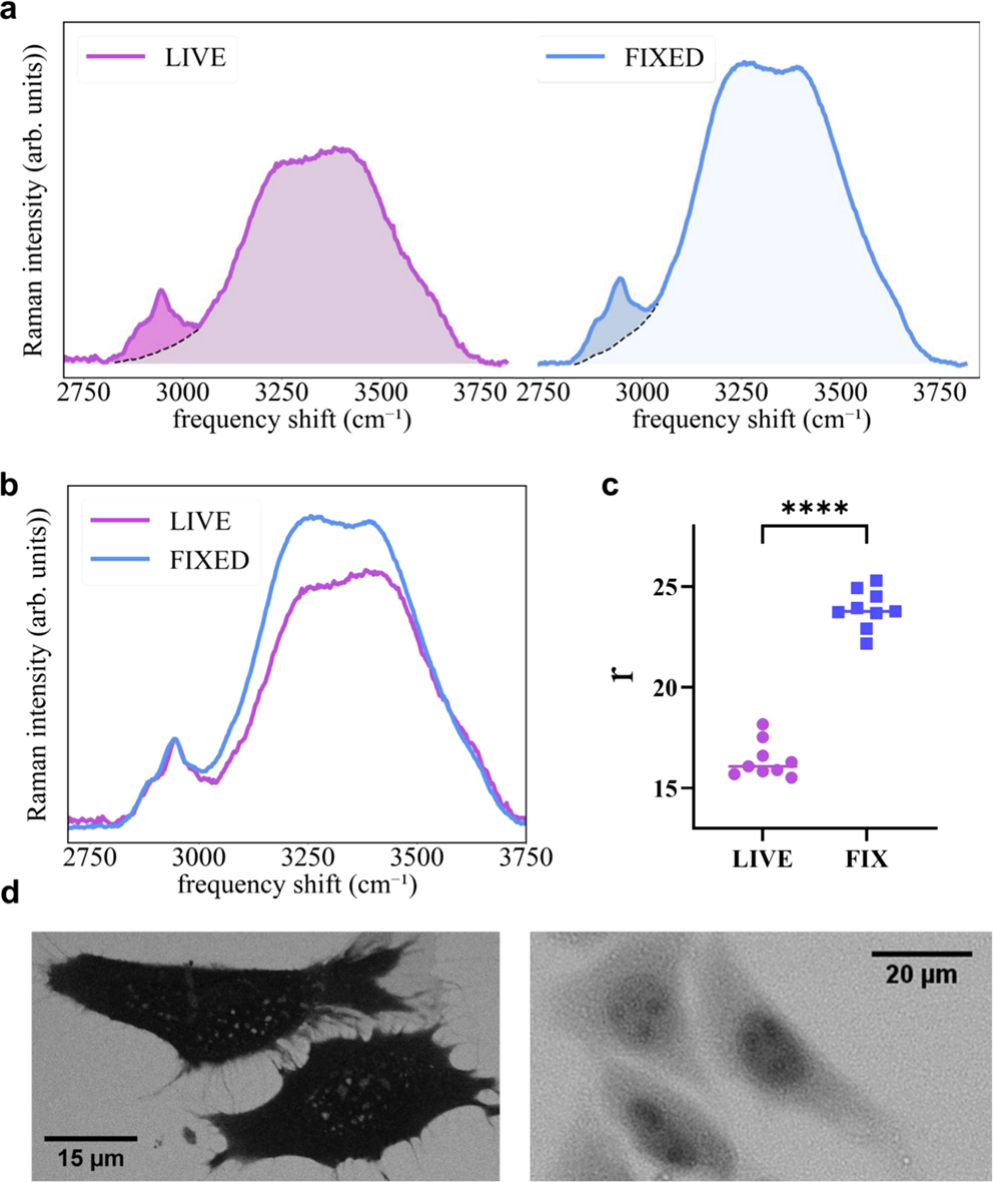
a) Representative high-frequency region of the Raman spectra for live and fixed cells. The areas of the two bands (CH—centered at 2900 cm^–1^ and OH—centered at 3300 cm^–1^) are filled with different colors. b) Spectra shown in a) normalized to the CH area. c) Scatter plot showing the ratio *r* between CH and OH band areas for each analyzed cell. The distributions show significant differences (unpaired two-tail *t*-test, *p*-value < 0.001). d) Representative fluorescence images acquired using inverse staining for live (left) and fixed (right) cells. The varying contrast between the cell and its surroundings indicates that fluorescein can penetrate the cell after fixation.

The relative intensities of the CH and OH stretching bands undergo substantial changes after fixation, as shown in Figure 2b. By normalizing the spectra to the CH band area, a significant increase in the OH band intensity appears. To quantify these changes, we studied the parameter *r,* defined as the ratio of the areas of the OH band to the CH band (Figure 2c). During the polymerization process triggered by paraformaldehyde, new covalent bonds are formed between the biomolecules, stabilizing the structure. The total number of CH bonds is not expected to change substantially^28^ or—at most—slightly increase,^29^ leading to a limited decrease of *r*. Hence, the pronounced increase in *r* obtained after fixation can only be associated with an increase in water content. In fact, recent studies have reported both a reduction in cell dry mass^30^ and a permeabilization of the plasma membrane following fixation.^31^ Consistently, this hypothesis is confirmed by the negative staining images presented in Figure 2d. Live cells (left panel) appear dark, as the dye (fluorescein) cannot pass through the membrane. In contrast, the cells appear much lighter after fixation, clearly indicating the loss of membrane integrity and the resulting increased permeability to water (free water) and small molecules.

Raman data provide a quantitative assessment of the variations in chemical composition, since the ratio *r* of the OH vs CH band areas is proportional to the mass fraction *w* of dry component and water within the scattering volume, multiplied by ϵ, the scattering efficiency per mass unit of CH and OH molecular stretching vibrations:

3 $$r=\frac{w_{OH}\epsilon_{OH}}{w_{CH}\epsilon_{CH}}$$

Assuming that the average scattering efficiency of the CH and OH bonds remains unchanged, given the limited number of bonds involved in PFA fixation,^28^ the relative increase in water content after fixation can be quantified as (Figure 2c):

4 $$R=\frac{r_{fix}}{r_{live}}=\frac{\overset{―}{w_{OH}}\epsilon_{OH}}{\overset{―}{w_{CH}}\epsilon_{CH}}{\left(\frac{w_{OH}\epsilon_{OH}}{w_{CH}\epsilon_{CH}}\right)}^{-1}=\frac{\overset{―}{w_{OH}}}{\overset{―}{w_{CH}}}\frac{w_{CH}}{w_{OH}}$$

where *w*_*CH*_ and *w*_*OH*_, $\overset{―}{w_{CH}}$ and $\overset{―}{w_{OH}}$ represent the mass fractions of the indicated chemical species for live and fixed cells, respectively.

The new amount of water mass fraction in fixed cells is

5 $$\overset{―}{w_{OH}}=R\frac{w_{OH}\overset{―}{w_{CH}}}{w_{CH}}$$

Assuming *w*_*OH*_ = 0.70 as the fraction of water mass in the live cell (and *w*_*CH*_ = 0.30 representing the fraction of dry mass)^32^ we estimate that after fixation $\overset{―}{w_{OH}}=0.77$ and $\overset{―}{w_{CH}}=0.23$ . In the following, we discuss how the observed variations in the local chemical composition directly influence the refractive index, the density, and, finally, the mechanical properties of the samples.

### 2.2 Density and Refractive Index

Modeling the cells as a “biphasic” medium composed of dry mass and water, the density of the cell can be written as a linear combination of the density of water and dry mass, weighted by their volume fraction Φ_i_:

6 $$\rho_{mix}=\Phi_{OH}\rho_{Water}+\Phi_{CH}\rho_{DryMass}$$

The volume fraction Φ_i_ of the *i*th component can be obtained from its mass fraction *w*_*i*_ and its density ρ_*i*_, given the density of the mixture ρ_*mix*_: Φ_*i*_ = *w*_*i*_ρ_*mix*_/ρ_*i*_.

In particular, for living cells, using the mass fractions derived from Raman data and a density of ρ_live_ = 1.08 g/cm^3^,^33,34^ the density of the dry mass is calculated as ρ_*DryMass*_ = 1.33 g/cm^3^. This value is in good agreement with that obtained for cells in suspension by measuring their buoyant mass in fluids based on H_2_O and D_2_O.^35^

Using this value, along with the same considerations for fixed cells, we found that the density of the cells after fixation decreases to ρ_*fixed*_ = 1.06 g/cm^3^, leading to a 2% change in the density. While the damage to cell membrane integrity after fixation has already been reported in several studies^30^ quantitative analysis of the mass density decrease at the single cell level is still rare. Here, we are proposing a new, fast, and contactless method for this evaluation, and the result is in agreement with a study performed using Surface Plasmon Resonance Imaging, which found a comparable mass density decrease after fixation.^31^

Changes in composition also result in variations in the refractive index. This parameter is determined for both live and fixed cells using phase holographic imaging by measuring the optical path difference (OPD), and subsequently through Raman-derived parameters used in the Lorentz–Lorenz formula. The maximum value of OPD (Figure 3a–c) is measured by probing the central part of cells, and it is significantly smaller in fixed cells. Since the OPD depends on the refractive index mismatch throughout the sample thickness *d* (see Methods, eq 10), this morphological parameter is needed to evaluate *n*. It was measured by performing *Z*-stacks with fluorescence imaging (Figure 3d,e), as well as analyzing Brillouin data while performing *Z*-scans along the cell.^36^ The data confirm that the fixation protocol does not significantly modify the cell thickness (Figure 3f). Thus, the significant variation measured in OPD (Figure 3c) can be completely ascribed to refractive index modifications. Considering the value of *n*_*medium*_ = 1.3370 ± 0.0001 for the buffer solution^37^ we obtained *n*_*live*_ = 1.374 ± 0.006 and *n*_*fixed*_ = 1.363 ± 0.003 (mean ± SD), finding a relative decrease after fixation of about 1%. This result is in agreement with the general trend reported for different cell lines by three-dimensional quantitative phase imaging, where a ubiquitous reduction of refractive index is associated with paraformaldehyde fixation.^38^

**Figure 3:**
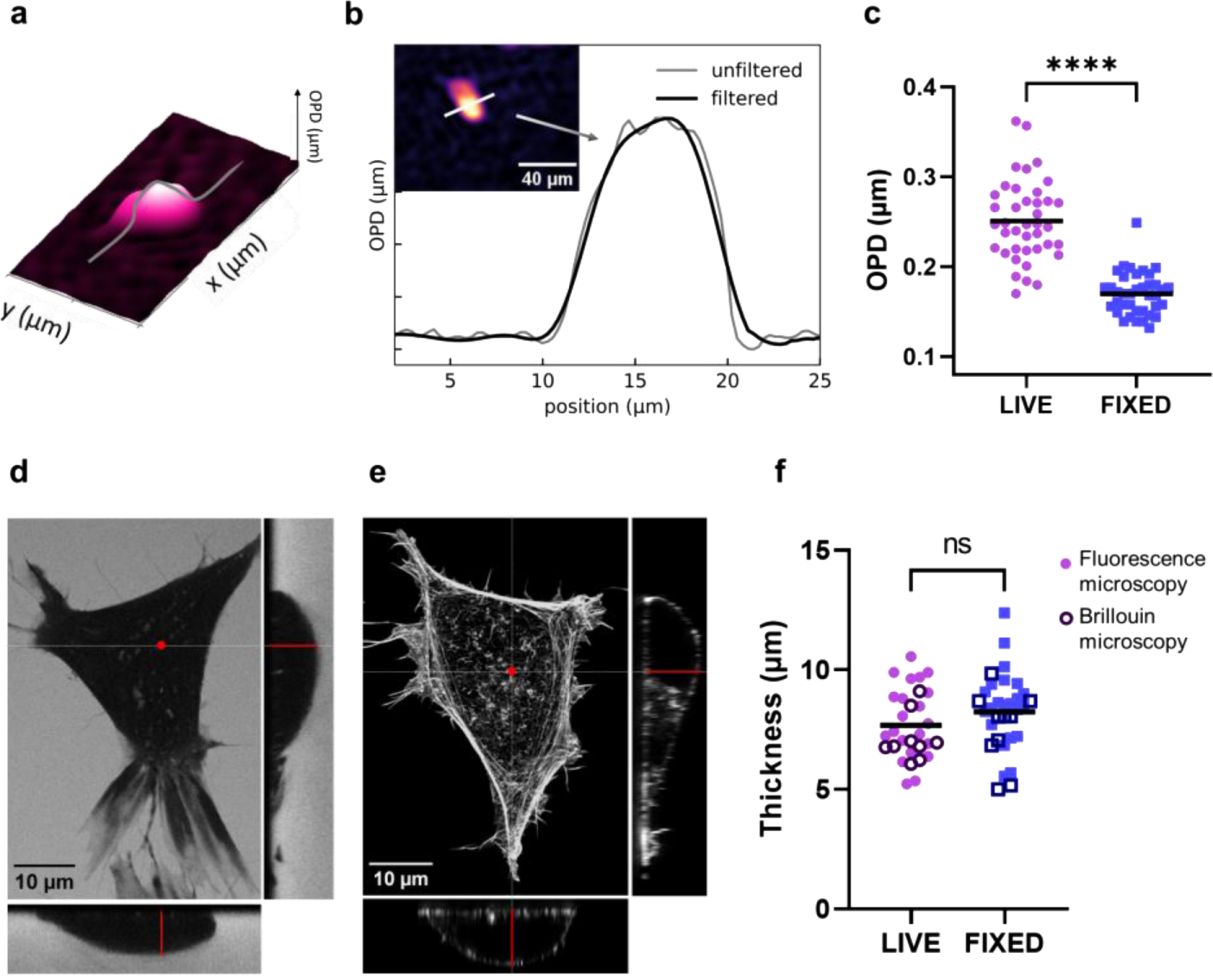
a) Representative image acquired with phase holographic imaging after removal of high-frequency component by FFT. The gray line indicates where the cell has been profiled. b) Cell profile obtained by plotting the grayscale value. Respectively, in gray and black, the profile before and after the noise removal. c) Scatter plot showing maximum OPD for each cell in the two cases. Distributions present significant differences (unpaired two-tail *t*-test, *p*-value <0.001). d, e) Representative orthogonal view of *z*-stacks acquired by fluorescence microscopy for live and fixed cells. f) Scatter plot showing the maximum thickness of each cell. Measurements have been acquired with fluorescence microscopy (full dots) and Brillouin spectroscopy (empty dots).

It should be highlighted that the decrease in the refractive index can be fully explained by the change in water content. In fact, by modeling the cell as a mixture of water and dry mass, we can use the Lorentz–Lorentz relationship to describe the dependence of the refractive index of a mixture as a function of the volume fractions of its components and their refractive indices.^39^

The Lorentz–Lorenz reads:

7 $$\frac{n_{mix}^{2}-1}{n_{mix}^{2}+2}=\Phi_{OH}\frac{n_{Water}^{2}-1}{n_{Water}^{2}+2}+\Phi_{CH}\frac{n_{DryMass}^{2}-1}{n_{DryMass}^{2}+2}$$

The refractive index of the dry mass, *n*_*DryMass*_, can be determined by combining the experimental value of *n*_*live*_ with the volume fractions Φ_*OH*_ and Φ_*CH*_, and applying eq 7. The resulting value, *n*_*DryMass*_ = 1.507 ± 0.006, lies within the range of refractive indices for mitochondria (1.400–1.420) and lysosomes (1.600).^40^ This value, when used in eq 7, makes it possible to track the evolution of the refractive index as a function of the water volume fraction.

The behavior is reported in Figure 4a and highlights that when the water content reaches the characteristic value of fixed cells $\overset{―}{\Phi_{OH}}=0.82$ (dashed vertical line in the figure), the refractive index expected by eq 7 is $n_{fixed}^{th}=1.364\pm 0.001$ . This calculated value aligns remarkably well with the refractive index of fixed cells previously measured by holography (*n*_*fixed*_ = 1.363 ± 0.003) (blue dot). The agreement, confirmed through independent measurements, validates the accuracy of the water volume fraction estimated via Raman spectroscopy and demonstrates that the modifications in sample composition account for the observed changes in refractive index.

**Figure 4:**
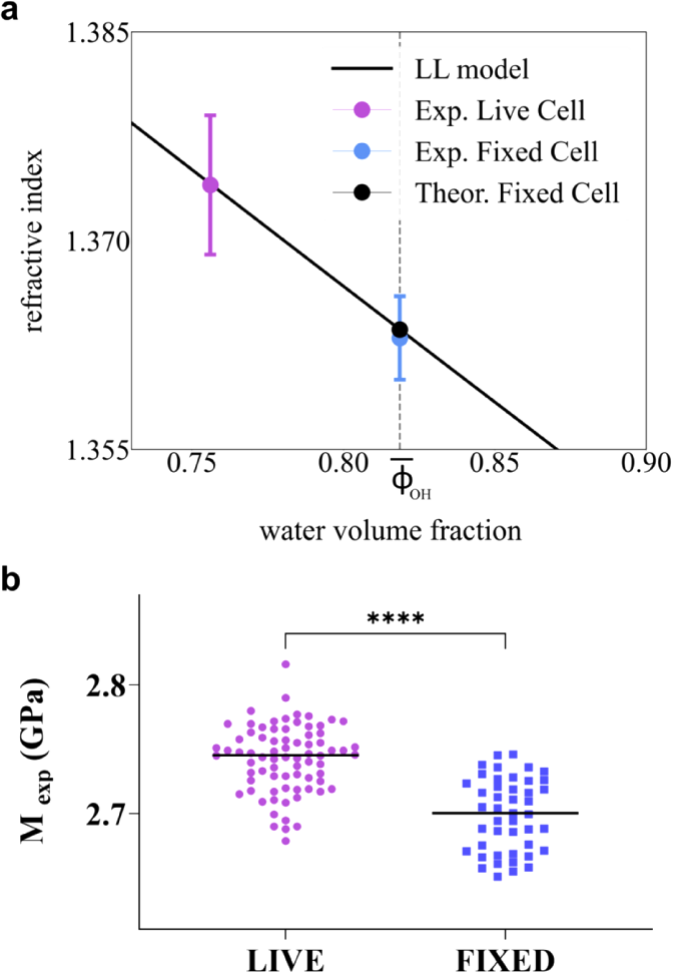
a) Summary of the obtained result for refractive index: the purple dot and blue dashed line represent the experimental value found for live and fix refractive index. Black solid line: Lorenz–Lorentz (LL) model as a function of the water volume fraction. Black dot: theoretical value of the refractive index of fixed cells evaluated from LL model. b) Scatter plot showing the longitudinal modulus evaluated in each cell. Distributions are statistically different (unpaired two-tail *t*-test, *p*-value < 0.001).

### 2.3 Determination of the Longitudinal Elastic Modulus

Using the values of *n*_*fixed*_, *n*_*live*_, ρ_*fixed*_, and ρ_*live*_, we can finally evaluate the longitudinal elastic modulus, *M*, from the Brillouin frequency shift. *M* confirms a reduction after fixation, as shown in Figure 4b—since the change of *n* ^2^/ρ (<0.3%, see Table 1) is negligible and cannot explain the modification of the Brillouin frequency shift. The significant softening after fixation seems to contradict AFM findings, which consistently report a notable increase of the Young’s modulus, *E*.^41,42^ This apparent discrepancy can be explained by the differing behavior of water during the measurement of *E* and *M*. In fact, while *M* measures the uniaxial strain response of the system to a uniaxial stress with transverse constraints, implying a volume-changing deformation, *E* is measured allowing for transverse expansion. In liquid-like material, and henceforth in water, this is reflected in a low value of *E* (∼kPa), while retaining values of *M* comparable with solids (∼GPa). Consistently, the amount of hydration is expected to massively impact the measurement of *M*, (*M*_*water*_ = 2.4 GPa) and be negligible for *E*.

**Table 1:** Summary of the Experimental Data for Refractive Index, Density, and Longitudinal Modulus for Living and Fixed Cells, Together with the Extrapolated Values for the Dry Mass

Samples	n	ρ [g/cm^3^]	*n*^2^/ρ [cm^3^/g]	*M*_*exp*_ [GPa]	*M*_*DryMass*_ [GPa]
Live cell	1.374 ± 0.005	1.08 ^33,34^	1.748	2.74 ± 0.03	-
Fixed cell	1.363 ± 0.003	1.06	1.753	2.70 ± 0.03	-
Liv. Dry Mass	1.507 ± 0.006	1.33	1.709	-	4.7 ± 0.4
Fix. Dry Mass	1.507 ± 0.006	1.33	1.709	-	5.8 ± 1.0

To disentangle the influence of the changed water content on the longitudinal elastic modulus before and after fixation, we further exploit the model of the cell as a biphasic system consisting of two noninteracting components: water and dry mass. The elastic modulus of the mixture is determined by the elastic moduli of these two components, weighted by their respective volume fractions. Two principal models have been proposed to describe this condition: the inverse rule of mixtures (Reuss model), which assumes an isostrain condition, and the direct rule of mixtures (Voigt model), applicable under isostress conditions. For example, according to the first model, which is also suitable for hydrated samples,^18,19,21^ the longitudinal modulus can be calculated as the sum of the dry mass and water components inversely weighted by their volume fractions:^18^

8 $$\frac{1}{M_{exp}}=\frac{\Phi_{OH}}{M_{Water}}+\frac{\Phi_{CH}}{M_{DryMass}}$$

The expected behavior of the longitudinal elastic modulus as a function of hydration level is reported in Figure 5a. For any given hydration, the elastic modulus of fixed samples (blue line) is always greater than that of the living ones (purple line), and only the differing water content in the two investigated conditions (filled points) can fully explain our findings. Interestingly, upon reaching the limit value Φ_*CH*_ = 1, it is possible to determine the changes in the longitudinal elastic modulus induced by fixation in the dry mass component, *M*_*DryMass*_.

**Figure 5:**
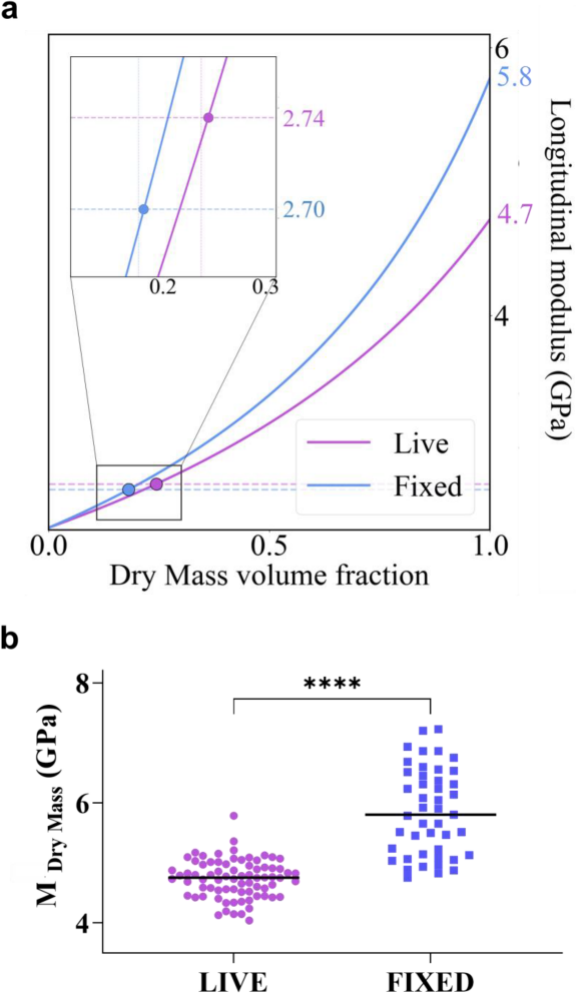
a) Summary of the obtained result for the longitudinal moduli evaluated with the Reuss model. b) Scatter plot showing the dry component of the longitudinal modulus evaluated using the Reuss model. The distribution is statistically different (unpaired two-tail *t*-test, *p*-value < 0.001).

We found that *M*_*DryMass*_ changes from 4.7 ± 0.4 GPa for live cells to 5.8 ± 1.0 GPa for fixed cells, with an overall increase of 21% (Figure 5a).

The substantial stiffening effect induced by paraformaldehyde in the dry mass is confirmed for all investigated cells (Figure 5b). This result aligns with the AFM measurements, considering that the dry mass is the component that plays the most important role in AFM measurements. Young’s modulus shows a 5-fold increase after fixation.^42^ Using the empirical scaling law observed by Scarcelli et al. in different biological systems^21^ a 5-fold increase in *E* corresponds to about a 14% increase in *M*. Although approximate, this estimation aligns with the value predicted by our analysis for the dry mass elastic modulus, ultimately achieving a quantitative reconciliation of the results obtained from the two techniques. While effective in the present case, this empirical law cannot be readily generalized to samples with markedly different elastic properties. Nevertheless, the ability of Raman spectroscopy to distinguish the contributions of different components in heterogeneous materials could support a more consistent application of the mixing rule for elastic moduli, thereby facilitating the comparison of results from experimental techniques that probe distinct moduli (e.g., Young’s modulus for indentation and OCE and longitudinal modulus for Brillouin microscopy) and operate at different frequencies.

## 3 Conclusions

In this study, we present a novel approach based on correlative Brillouin and Raman microspectroscopy (BRMS) to quantify the water content and its influence on the mechanical properties of a hydrated material.

The potential of the method has been demonstrated by studying the impact of fixation on cells, where the application of the proposed theoretical framework reconciled the apparent discrepancy observed with AFM results. Considering the cells as biphasic materials composed of fluid (water) and solid (dry mass) constituents, the increase in water content determined by Raman spectroscopy is at the origin of the reduction in density, refractive index, and longitudinal elastic modulus. Decoupling the water contribution, our method discloses the mechanical variation of the dry component, which suffers, as expected, significant stiffening, in agreement with several AFM-based findings. In conclusion, by integrating an innovative Raman data treatment and a deep Brillouin analysis, we uncovered a critical relationship between chemical composition and stiffness, in the highly debated context of highly hydrated samples.

This advancement provides a powerful tool for more accurate interpretation of Brillouin spectroscopy data in biological research and can be broadly applied across a range of biological processes.

## 4 Methods

### 4.1 Sample Preparation

For this investigation, the HEK293T cell line from ATCC (https://www.atcc.org catalog number: CRL-3216) was used. Cells were cultured as described in previous work^36,43^ in tissue culture polystyrene flasks in DMEM High Glucose (Euroclone S.p.A.) supplemented with 10% fetal bovine serum (Euroclone S.p.A.), 1% penicillin–streptomycin (Euroclone S.p.A.), and 2 mM l-glutamine (Euroclone S.p.A.) in a humidified atmosphere with 5% CO_2_ at 37^◦^C. The medium was changed every 3 days.

Samples were prepared similarly for Brillouin, Raman, Fluorescence, and Holographic measurements. Cells were trypsinized, seeded at a concentration of 5 × 10^3^ cells/mL in glass-bottom Petri dishes, and cultured in the growth medium. Three glass dishes were used for live cell analysis, and the other three were fixed with 4% paraformaldehyde for 20 min at room temperature and washed with PBS. During Brillouin and Raman measurements, cells, both live and fixed, were maintained in the cell media and maintained into an incubator chamber (UNO-T-H-PREMIXED—OKOLAB) at 37 ^◦^C in controlled humidity and CO_2_ conditions (5% CO_2_). For fixed samples, measurements were performed within a week from fixation. For holographic measurements, the dishes were inserted in the microscope incubator (37^◦^C and 5% CO_2_ concentration) and covered with Hololids, lids designed to eliminate image disturbances caused by condensation and surface vibrations, while maintaining optimal air ventilation.

For the inverse fluorescence experiment, fluorescein powder (Fluka) was diluted in the cell medium at a concentration of 1.5 mg in 7 mL and added to the sample. Fluorescein stained the medium without entering the cell. Instead, fixed samples were permeabilized with 0.1% Triton X-100 for 5 min, washed with PBS, and then blocked for 30 min in 1% bovine serum albumin (BSA). Alexa Fluor 488 Phalloidin (Thermo Fisher Scientific, Waltham, MA, USA), diluted appropriately (1/250 in 1% BSA), was added to stain F-actin, and the samples were incubated in the dark for 1 h. Afterward, they were washed twice with 0.5% Tween-20 and mounted onto glass slides using a VECTASHIELD mounting medium (Vector Laboratories).

### 4.2 Spectroscopic Characterization and Data Analysis

Spectroscopic measurements were performed using the correlative Raman-Brillouin setup built in the GHOST Lab in Perugia (Italy).^22^ The acquisition system has been equipped with a custom-made inverted microscope, where a λ = 532 nm laser beam (Spectra Physics Excelsior) with a power of 15 mW is used as a light source. A microscope objective UPLSAPO 60XW (Olympus, NA = 1.2) is used to focus and collect the backscattered light. The deeply inelastic scattered light is sent to the Raman spectrometer (Horiba iHR320 Triax), while the quasi-elastic component enters the Brillouin TFP-2 HC interferometer. For the Brillouin measurements, the achieved spatial resolution is ∼1 μm and ∼4 μm in the lateral and axial directions, respectively. After focusing the laser beam into the sample, linear scans were performed along the central axis of several cells (*N* = 10 and *N* = 17 for fixed and living cells, respectively). In order to acquire information from all the subcellular structures (nucleus, cytoplasm, and nucleoli), a step size of 1.5 μm was chosen for the measurements to find a compromise between resolution and acquisition speed. Brillouin spectra were fitted using a damped harmonic oscillator (DHO) function convoluted with the instrument response function *R*(ω):^11^

9 $$I\left(\omega \right)=\frac{I_{0}}{\pi }\frac{\Gamma \omega_{B}^{2}}{{\left(\omega^{2}-\omega_{B}^{2}\right)}^{2}+\Gamma^{2}\omega_{B}^{2}}⊗R\left(\omega \right)$$

The fitting parameters ω_*B*_, Γ, and *I*_0_ correspond to the Brillouin frequency shift, peak width, and intensity, respectively. The response function takes into account the peak broadening induced by the finite resolution of the FP spectrometer (about 100 MHz) and the spread in the exchange wavevector (**q**) induced by the use of a high NA objective.^11^ To determine the cell thickness, *z*-axis scans were performed, and the cell interfaces were identified by analyzing the behavior of the Brillouin fitting parameters, as reported in Ref. [^36^].

The high-frequency region of the Raman spectra was analyzed, focusing on the OH stretching band of water centered around 3300 cm^–1^, and the CH stretching band centered around 2900 cm^–1^.^26^ A spline was used to subtract the background from the spectra, and the spectrum acquired from the buffer solution was used to subtract the OH band and isolate the contribution of the CH stretching band. The two spectra were normalized to the maximum intensity of the OH band prior to subtraction. Finally, the areas of the two bands were evaluated.

### 4.3 Holographic Microscopy

Phase holographic images were acquired using a Holomonitor live cell imaging system (Phi). For each point in the sample, 5 pictures acquiring the optical path difference (OPD) were taken every 10 min; the more focused one was saved as TIFF images and analyzed using the free software Gwyddion. In order to eliminate the noise background, we removed the higher frequency components of the Fast Fourier Transform (FFT), which were the biggest contributors to the background noise. The obtained image quality was good enough to extract the cell profiles, from which, in turn, we extracted the maximum OPD value for each cell, which is related to the cell thickness through the refractive index:

10 $$OPD=d\left(n_{cell}-n_{medium}\right)$$

with *d* being the sample thickness. After eliminating the noise background, we got a 2D image for every measurement, where the grayscale value of each pixel represented the OPD at that position.

### 4.4 Fluorescence Microscopy

Fluorescence 3D images were acquired with the LSM900 with Airyscan 2 (ZEISS), using a 40x Plan-Apochromat oil immersion objective with 1.3 NA. The sample was excited using a 493 nm laser, and the emitted light was collected using the Airyscan sensor.^44^ *Z*-stacks were acquired for each cell. A step size of 0.038 μ*m* was chosen for *x* and *y* axes, while for *z*, we opted for the recommended parameter for Airyscan processing, which was 170 nm, to achieve super-resolution images and minimize errors. With these parameters, we achieved a planar resolution (*XY*) of 120 nm and an axial resolution (*Z*) of 350 nm. The determination of the thickness through fluorescence imaging employed two different methods. For the fixed sample, actin fibers were stained in order to reconstruct the 3D structure of the cell. For the live sample, instead, we introduced fluorescein into the cell media. This nontoxic substance emits light when excited by light (peak excitation wavelength ∼480 nm, peak emission wavelength ∼520 nm) and it is commonly employed as a fluorescent tracer.^45^ It dissolves in the medium without penetrating inside the cell. Consequently, in the confocal images, the fluorescence signal comes from the solution, while the cells appear as dark voids.^46^

We used Fiji software for the analysis^47^ working with the orthogonal planes of each z-stack to identify the cell shape and thickness. In particular, for live samples, we estimated thickness by measuring the dark void, corresponding to the cell, where fluorescein cannot penetrate. In contrast, for fixed samples, the shape of the cell was determined by looking at the actin fibers present in the cell cytoplasm. To minimize error, measurements were repeated 6 times for each picture; the mean and SD were used, respectively, as the final value and error. The thickness of the cell was acquired on both *XZ* and *YZ* planes, and to be coherent to the phase holographic analysis, it was measured at the highest part of the cell.
